# Development and application of biogas project for domestic sewage treatment in rural China: opportunities and challenges

**DOI:** 10.2166/washdev.2017.011

**Published:** 2017-10-20

**Authors:** Shikun Cheng, Mingyue Zhao, Heinz-Peter Mang, Xiaoqin Zhou, Zifu Li

**Affiliations:** 1 School of Energy and Environmental Engineering, Beijing Key Laboratory of Resource-Oriented Treatment of Industrial Pollutants, University of Science and Technology Beijing, Beijing 100083, China; 2 Beijing Municipal Environmental Monitoring Center, Beijing 100048, China

**Keywords:** biogas, challenge, domestic sewage, opportunity, rural China

## Abstract

The biogas project for domestic sewage treatment (BPDST) is considered a promising facility for wastewater management in rural areas of China. This paper explores previous experimental works, cost analysis, and BPDST structure and design based on Chinese literature. Opportunities for developing decentralized or neighborhood-based BPDSTs include fulfilling Millennium Development Goals (MDGs) and Sustainable Development Goals (SDGs), the water pollution situation and deficiency of wastewater treatment facilities, the advantages of BPDSTs compared with centralized sewage plant, government support and policy drive for rural wastewater treatment, and reuse demand for resources. Meanwhile, challenges faced are emphasized as follows: uncertain responsibility for BPDSTs under different governmental departments restricts BPDST development and should be specified; uncertain effluent quality due to low efficiency of nutrient removal requires aerobic post-treatment to some extent; rural environmental awareness is still low and should be heightened; more funds should be invested in R&D for improvement of technology innovation; more reuse and resource recovery elements should be considered during implementation; follow-up services are lacking and should be improved; and BPDST maintenance should be trained. This paper could provide valuable reference for other developing countries.

## INTRODUCTION

Domestic sewage and fecal sludge in rural areas pose a risk to the health of people and restrict the well-being of rural inhabitants not only in China but throughout the world (Strande et al. 2014; Mills & Cumming 2016). At present, only 11.4% of wastewater is managed and treated in the villages of China, against an over 91.9% treatment rate in urban areas (MOHURD 2016). The number of wastewater treatment plants (WWTPs) increased from 763 in 2007 to 3,437 in 2015 at the level of town and township, which are considered parts of rural areas of China. However, many WWTPs in rural areas have not operated because of incomplete drainage networks. In addition, centralized wastewater treatment systems are costly to build and maintain (Giovanni et al. 2012), especially in rural China with low population densities and dispersed households. Alternatively, the decentralized approach for wastewater treatment, which employs a combination of onsite and unpowered/ low-powered cluster systems, is attracting increasing attention (May et al. 2009; Aditi & Sorada 2011). Anaerobic treatment combined with biogas production is a promising technology for domestic sewage treatment in rural China ( Jiang et al. 2011; Cheng et al. 2013). This technique has been evaluated as one of the most energy-efficient and environmentally beneficial technologies for bioenergy production (Bond & Templeton 2011).

The dominant biogas digester in rural areas mainly aims to treat animal waste and is integrated in livestock and poultry breeding and agriculture production systems. However, another mainstream type of biogas digester, the domestic sewage digester (DSD), is applied to treat domestic sewage (Cheng et al. 2014). DSD was developed in the 1980s; in view of sewage characteristics, the household-scale biogas digester replaces a septic tank to treat domestic sewage and fecal sludge. Rural energy departments at all levels began to promote DSD in rural China (Zheng et al. 2002). By 2015, 30.52 million toilets were connected to DSDs in China (NHFPC 2016).

DSD is the core of any biogas project for domestic sewage treatment (BPDST), which is a type of decentralized wastewater technology. BPDST is suitable for places where sewer pipeline systems are unavailable and serves residential buildings, office buildings, hotels, schools, public toilets, and hospitals. Table 1 presents the number of BPDSTs in recent years. The number of decentralized BPDSTs reached its peak in 2013. An increasing number of villages and townships started to construct centralized WWTPs due to the expansion of urbanization; thus, more wastewater is collected in WWTPs via sewer pipeline net-works. As a result, the number of BPDSTs is decreasing, and this trend will continue. However, the rate of decrease is not high, and BPDSTs will still function in rural areas in the long run. In China, BPDST is more popular in Sichuan, Jiangsu, and Zhejiang Provinces due to the high disposal rate of rural domestic sewage in these provinces. These regions are located in the south of China, where the climate is considerably suitable for anaerobic digestion. In addition, local governments emphasized rural wastewater management and provided preferential policies and guidelines for such projects. Internationally, similar systems are also developed and introduced as decentralized wastewater treatment systems (DEWATS) or decentralized sanitation and reuse (DESAR).

**Table 1:** Statistics of BPDSTs in China in recent years (2009–2015)

YEAR	2009	2010	2011	2012	2013	2014	2015
Number of existing BPDSTs	186,945	191,613	198,347	208,551	213,200	210,719	202,039
Accumulated volume of all digesters (104 m3)	851.4	894.2	930.1	970.0	1,009.7	–	–
Category							
Village	46,153	57,053	68,509	72,700	78,800	–	–
School	6,237	6,927	7,253	7,368	7,471	–	–
Others	134,555	127,633	122,579	128,483	127,000	–	–

Sources: Ministry of Agriculture (MOA 2015); *2015 China Rural Statistical Yearbook* (NBS 2015); *2016 China Rural Statistical Yearbook* (NBS 2016).

BPDST has numerous advantages, such as easy onsite construction, minimal land occupation, easy maintenance, low energy input, and good environmental benefits. BPDST has been promoted in rural China in recent decades. However, the situation of BPDST is not at the optimum level. This paper mainly reviews BPDST technology by presenting the opportunities for its development in rural China and discussing its many impediments and challenges.

## CHINESE LITERATURE REVIEW

Previous studies and evaluation of BPDST have mainly focused on the hygiene effect of pathogen removal by DSD (Wu & Xu 2003). A study by Sichuan Province Institute of Parasitic Disease Prevention and Control tested the BPDST treatment effect in six projects. Generally, the quality of treated sewage improved considerably after 1–4 years of operation. Thermotolerant coliforms are >10^—4^. The number of parasitic ova ranges from 0.565/L to 1.074/L; biochemical oxygen demand (BOD) is <50 mg/L; suspended solids (SS) is <60 mg/L; color is <100. These indicators could meet the requirement of Integrated Wastewater Discharge Standard (GB8978-2002) and Sanitary Standard for the Non-hazardous Treatment of Night Soil (GB7959-1987; Zheng et al. 2006). Normally, BPDST can remove chemical oxygen demand (COD) and BOD_5_ by 74–90% and 80–90%, respectively (Xie et al. 2005). Zhao et al. reported the operation situation of six BPDSTs in different buildings and summarized the digester shape, digester volume, and design key parameters (Zhao et al. 1996). Zang et al. compared the function of different packings and fillers and suggested that half-soft packing and polyurethane foam board soft filler are applied into the anaerobic zone and post-treatment zone, respectively (Zeng et al. 1998). Zang et al. (2012) compared treatment effects for rural wastewater by stabilization pond and BPDST in terms of pH, total nitrogen (TN), total phosphorus (TP), ammonia-nitrogen (NH_4_^+^-N), BOD_5_, COD, SS, and color. Except for pH value, BPDST showed high removal rates in all indexes (Zang et al. 2012). In addition to hygiene effect and pollutant removal, BPDST can also generate biogas for cooking. Some literature indicated that the biogas produced from the wastewater of 10–12 house-holds can provide one household with cooking fuel.

A large-scale survey on effluent from BPDST was conducted during 1998–2000 in Sichuan Province (Tian et al. 2002). A total of 88 BPDSTs were sampled randomly. Among the 88 BPDSTs, 55 BPDSTs were installed at office buildings and 33 BPDSTs served at dormitory buildings. Continuous 3-year monitoring showed that NH_4_^+^-N, SS, and pH qualified rates of effluent (to meet Grade 1 level) were all above 86.4%; COD, BOD_5_, and the rmotolerant coliforms qualified rates of effluent (to meet Grade 1 level) were between 77.3 and 40.9%, whereas odor and color were below 55% (see Table 2). BPDST hygiene effect and qualified effluent in dormitory buildings and office buildings showed no obvious difference (see Table 3). How-ever, the treatment effect during spring and summer was better than in winter and autumn (see Table 4). Although anaerobic systems do not normally achieve ammonia removal, an NH_4_^+^-N concentration of 20 mg/l could be achieved because of the inflow dilution at a low concentration level. Generally, low values of NH_4_^+^-N are only possible after adequate aerobic post-treatment. Further analysis cannot be made because the incoming concentrations are unavailable from this survey. Nevertheless, the results are optimistic based on the large-scale investigations which were published 15 years ago by literature retrieval; however, their present function is unknown. According to the onsite observations of authors in recent years, some BPDSTs fail without correct long-term operation, which will be discussed in the subsequent sections of this paper.

**Table 2:** Statistical results of 88 BPDSTs’ effluent in 1998–2000

	1998			1999			2000		
		Qualified rate^a^ (%)			Qualified rate (%)			Qualified rate (%)	
	Average	G1	G2	Average	G1	G2	Average	G1	G2
COD, mg·L^−1^	152	75.0	82.1	174	60.5	89.5	240	40.9	59.1
BOD5, mg·L^−1^	81	71.4	89.3	84	73.3	94.7	94	77.3	86.4
NH_4_^+^-N, mg·L^−1^	20	100	100	32	94.7	100	24	100	100
SS, mg·L^−1^	43	96.4	96.4	33	94.7	100	46	90.9	100
Color, degree	60	14.3	42.9	40	26.3	86.6	35	40.9	90.9
pH	7	96.4	96.4	7	92.1	92.1	7	86.4	86.4
Odor/grade	–	53.6	96.4	–	31.6	100	–	31.8	77.3
Thermotolerant coliforms^b^	–	60.7	60.7	–	47.4	47.4	–	72.7	72.7
Parasitic ovum/L	0	100	100	0.55	97.4	97.1	0	100	100

Notes: G1 = Grade 1; G2 = Grade 2. The effluent standards for G1 are: COD ≤ 200, BOD5 ≤ 100, NH_4_^+^-N ≤ 70, SS ≤ 100, color ≤ 30, odor ≤ 2, thermotolerant coliforms ≥10^−4^, parasitic ovum≤3, pH = 7–8; G2 are: COD ≤ 300, BOD5 ≤ 150, NH_4_^+^-N ≤ 100, SS ≤ 150, color ≤ 40, odor ≤ 4, thermotolerant coliforms ≥10^−4^, parasitic ovum ≤3, pH = 7–8 (refer to above table for units), according to local standard DB51/136-92 *Effluent Hygiene Standard from Domestic Sewage Purification Biogas Digester*.

a Qualified rate = number of BPDSTs that meets certain index (COD, BOD5, NH_4_^+^-N, etc.)/total sample number (88 in this paper).

b Testing of thermotolerant coliforms adopts the Chinese test standard similar to the multi-tube fermentation method. The unit refers to the minimum sample volume to identify a thermotolerantcoliform colony. For example, 10^−4^ indicates that the minimum sample volume to test a thermotolerant coliform colony is 10^−4^ L = 0.1 mL.

**Table 3:** Statistical results of BPDST effluent from different sources

	Office building (*n* = 55)		Dormitory building (*n* = 33)	
	G1 qualified no. (rate, %)	G2 qualified no. (rate, %)	G1 qualified no. (rate, %)	G2 qualified no. (rate, %)
COD, mg·L^−1^	32 (58.2)	46 (83.6)	21 (63.6)	24 (72.7)
BOD_5_, mg·L^−1^	43 (78.2)	51 (92.7)	22 (66.7)	32 (87.8)
NH_4_^+^-N, mg·L^−1^	54 (98.2)	55 (100)	33 (100)	33 (100)
SS, mg·L^−1^	52 (94.5)	54 (98.2)	31 (93.9)	33 (100)
Color, degree	19 (30.9)	42 (76.4)	6 (18.2)	23 (69.7)
pH	53 (96.4)	53 (96.4)	28 (81.8)	28 (81.8)
Odor (grade)	22 (59.5)	37 (100)	19 (65.5)	28 (96.6)
Thermotolerant coliforms	34 (61.8)	34 (61.8)	17 (51.5)	17 (51.5)
Parasitic ovum/L	55 (100)	55 (100)	32 (96.9)	32 (96.9)

**Table 4:** Statistical results of BPDST effluent in different seasons

	Spring and summer (*n* = 28)		Autumn and winter (*n* = 60)	
	G1 qualified no. (rate, %)	G2 qualified no. (rate, %)	G1 qualified no. (rate, %)	G2 qualified no. (rate, %)
COD, mg·L^−1^	21 (75.0)	23 (82.1)	32 (53.3)	47 (78.3)
BOD_5_, mg·L^−1^	20 (71.4)	25 (89.3)	45 (75.0)	55 (91.7)
NH_4_^+^-N, mg·L^−1^	28 (100)	28 (100)	58 (96.7)	60 (100)
SS, mg·L^−1^	26 (96.4)	26 (96.4)	56 (93.3)	60 (100)
Color, degree	4 (14.2)	12 (42.9)	19 (31.7)	53 (83.3)
pH	27 (96.4)	27 (96.4)	54 (90.0)	60 (100)
Odor (grade)	15 (53.6)	27 (96.4)	26 (68.4)	38 (100)
Thermotolerant coliforms	16 (60.7)	16 (60.7)	34 (56.7)	34 (56.7)
Parasitic ovum/L	28 (100)	28 (100)	59 (98.3)	59 (98.3)

Qian et al. (2014) studied the cost of DSD–BPDST compared with other decentralized wastewater technologies (Table 5) and the Ministry of Environmental Protection (MEP) (2013) launched the Guideline on Project Construction and Investment for Rural Sewage Treatment. The construction cost of BPDST is relatively small, especially when the project capacity is large. However, the temperature will limit the normal BPDST function especially in winter because the core of BPDST adopts anaerobic technology. BPDST is best suited for east and south China, where the climate is relatively warm.

**Table 5:** Cost comparison of decentralized wastewater treatment projects in rural China

		Investment cost (CNY) per tonne wastewater by capacity				Operation cost (CNY)/t/day
	Technology	<1 m^3^/d	2–4 m^3^/d	< 5–9 m^3^/d	>10 m^3^/d	
1	Small-scale constructed wetland	2,800–3,700	2,600–3,300	2,600–3,200	2,300–2,900	<0.1
2	Land treatment	2,600–3,300	2,200–2,900	2,000–2,600	2,000–2,400	<0.2
3	Stabilization pond	2,300–3,300	2,300–2,600	2,000–2,400	1,900–2,400	<0.1
4	BPDST	2,600–5,200	2,600–3,900	1,900–3,300	600–2,000	<0.2
5	Small-scale integrated sewage treatment facility^a^	32,000–39,000	19,500–28,000	13,000–22,000	11,000–15,000	0.1–0.8

Source: Ministry of Environmental Protection 2013.

Note: 1 USD ≈ 6.87 CNY (Bank of China, January 25, 2017).

a Small-scale integrated sewage treatment facility can be considered as ‘mini WWTP’ but is prefabricated off-site. Such a facility consists of primary settling tank, biological treatment tank, secondary settling tank, sludge tank, etc. Normally, this facility adopts an aerobic process, such as biological contact oxidation tank with aeration; thus, it can also be regarded as a combination of activated sludge process and biofilm process.

## STRUCTURE AND DESIGN OF BPDST

**Figure 1:**
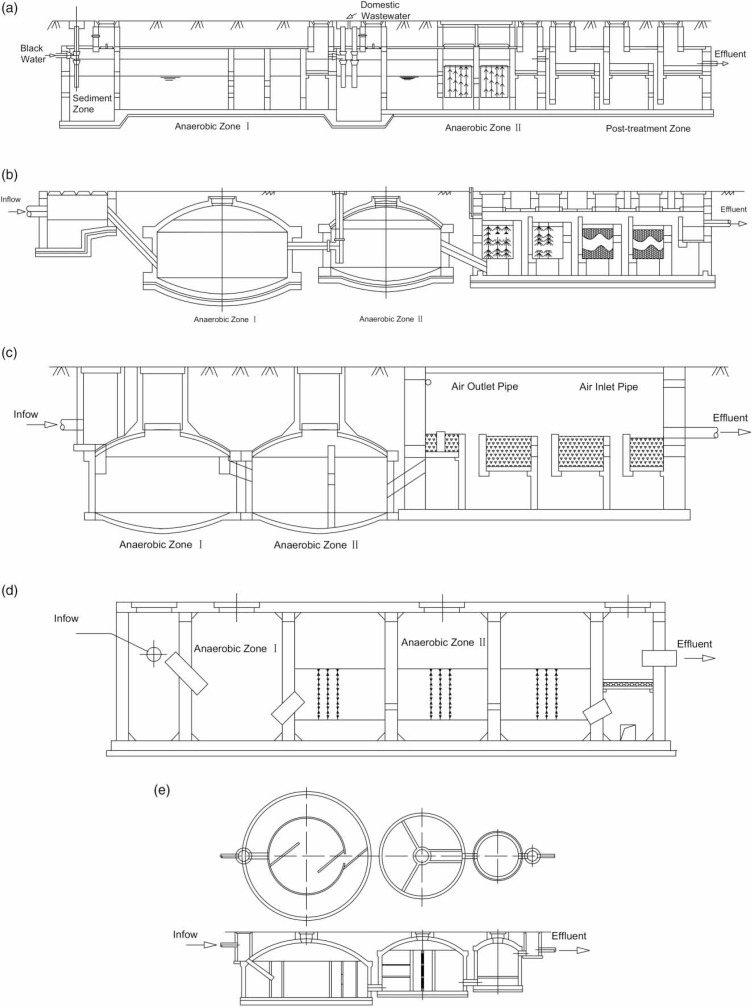
Structure of different BPDST types. (a) Type A: Sichuan Province, (b) Type B: Zhejiang Province, (c) Type C: Jiangsu Province, (d) Type D: Sichuan Province, (e) Type E: Sichuan Province.

In 1991, the Sichuan Rural Energy Office compiled the first drawing collection of BPDST, in which 10 types of BPDST were presented and some types were improved (Mao 2003). The digester can be strip-type, rectangular, or round (Xia 2007). In 2014, the Ministry of Agriculture (MOA) officially issued the professional standard for BPDST, entitled *Collection of Standard Design Drawings of Biogas Digester for Domestic Sewage Treatment*, NY/T 2597-2014 (MOA 2014a). This standard was the collection of provincial BPDST types. Generally, five representatives exist through-out China (Xia et al. 2008), as shown in Figure 1 and Table 6.

**Table 6:** Main parameters of the five representative digesters

					Percentage of each unit			
Type	HRT (hours)	Inflow type	Effective volume (m^3^)	Sediment tank	Sediment zone (%)	Anaerobic zone I (%)	Anaerobic zone II (%)	Post-treatment zone (%)
A	72	Separated	100	No	10.2	33.5	31.7	24.6
B	72	Combined	50	Yes	–	40.0	26.6	33.4
C	48–72	Combined	17	Yes	–	35.0	35.0	30.0
D	96	Combined	60	No	12.5	18.8	56.2	12.5
E	96	Combined	90	Yes	–	66.7	27.1	6.2

HRT, hydraulic residence time.

At present, BPDST is composed of a sediment tank or sediment zone, anaerobic zones I and II, and post-treatment zone. The sediment tank or sediment zone is used for removing non-biodegradable and large solid bodies, whereas anaerobic zone I digests organic pollutants. Soft packing is filled into anaerobic zone II as a microbe carrier for further degradation of organics. The post-treatment zone is installed with packing and filler, which also act as a filter. The two types of inflow system are separated inflow and combined inflow. The combined inflow type is better than the separated inflow type in terms of investment. However, the concept of source separation has become increasingly known and is encouraged by the government (Hu et al.^2016^). The separated inflow type meets the requirements for future development. Type A adopts separated inflow system and tunnel-type tanks, and the soft packing is filled into anaerobic zone II (see Figure 1). Type B fits the separated and combined inflow systems. A 10% gradient exists at the bottom of a sand sediment tank. An extra inflow hole is set in anaerobic zone II for other types of wastewater. A ventilation pipe is installed in post-aerobic treatment. Type C adopts two cylinder-shaped digesters, and a baffled wall is placed inside digester II. Post-treatment adopts a facultative biofilter, with the filler at the particle size of 5–40 mm and a layer 500 mm high. Structurally, two cylinder-shaped digesters and a rectangular biofilter are built separately and connected by a PVC pipe, which can address the phenomenon of uneven foundation settlement. Type D has tunnel-style tanks, and the entire system is maintained under anaerobic conditions. The post-treatment only performs as an outlet tank with filler plate. The inlet and outlet pipes in anaerobic zone II are set at a 45^o^ angle, which helps the inflow to stir the settling sludge. Type E consists of a series of three digesters. A backflow and baffled wall are set inside digester I. The inflow goes directly into inner concentric circles and an S-type flow to avoid short pass and extend the retention time. A triangular baffled wall is also set in digester II to realize intensive mixing. To summarize, types D and E adopt anaerobic technology that reduces the function of nitrogen and phosphorus removal.

The calculated ratio of functional and post-treatment units in types A, B, and C is 7.5:2.5, 2:1, and 7:3. Types D and E can almost reach 9:1. Based on this result, the future trend of designing BPDST is to reduce the volume of the post-treatment unit. This trend supports the function of soft package and microbe absorption in anaerobic zone II, which is beneficial to the degradation process of dis-solved organic matter. However, the function of the post-treatment zone is weakened and simplified, which may reduce the function of nitrogen and phosphorus removal. Hu et al. (2002) studied the contribution of each unit to COD removal. Approximately 90% of COD removal was achieved in sediment and anaerobic zones, whereas the facultative filtration zone only removed 10% of COD, even though it accounts for 45% of the volume of the entire system. When BPDST installs facultative filtration as post-treatment, the ventilation should be carefully considered. Such a pipe is similar to the ventilation pipe in the national standard septic tank (CIBSDR 2006).

## OPPORTUNITIES FOR DEVELOPING BPDST

### MDGs and SDGs

The world must first cross the water barrier to achieve the Millennium Development Goals (MDGs) (ADB et al. 2006). As the new and broader sustainability agenda, the Sustainable Development Goals (SDGs) extend further than the MDGs. By 2030, SDGs require improvements in water quality by reducing pollution, eliminating dumping and minimizing the release of hazardous chemicals and materials, halving the proportion of untreated wastewater, and substantially increasing recycling and safe reuse globally (UNESCAP et al. 2015).

Following the MDGs, the world, including China, is striving to meet the SDGs. The efficiency of wastewater treatment in rural areas plays a significant role in fulfilling the SDGs. Most people without access to safe drinking water and sanitation usually live in rural areas. In China alone, the population in rural areas that suffers from contaminated water sources counts at 298.10 million people (NDRC et al. 2012). Approximately 9 billion metric tonnes of domestic wastewater are discharged every year in rural areas of China (Zhou et al. 2008). The popularization of BPDST can increase the possibility of achieving some of the SDGs to a great extent. Increasing the accessibility to water and sanitation does not imply overexploitation of the existing resources but improving their management by reducing, recycling, and reusing as well as identifying new water sources, such as storm water and reclaimed waste-water (Giovanni et al. 2012).

## Water pollution and infrastructure deficiency

Environmental pollution transfers from urban areas to rural areas. In this case, infrastructure construction that aims at wastewater treatment in rural areas is significant to improve rural environmental quality. Rural areas do not usually have sewage systems; approximately 96% of villages lack drainage and wastewater treatment systems (Bai et al. 2008). By 2015, only 11.4% of wastewater was managed and treated in rural areas. Most of the domestic wastewater and runoff are dis-charged directly to the surface water. This situation has resulted in serious non-point pollution in many areas. In addition, the wastewater treatment facilities are largely simple and primary. Only septic tanks or biogas digesters are usually built. These primary treatments cannot guarantee the harmless disposal of wastewater and the achievement of the required effluent quality (Wang & Zhang 2011).

The absence of and defects in infrastructure create demand for sewage digesters because the problems and limitations of the centralized approaches for wastewater treatment are progressively surfacing. Rural areas lack the funding to construct centralized facilities and the technical expertise to manage and operate them. Alternatively, a sewage digester allows for flexibility in management, and simple as well as complex technologies are available. Sewage digesters are ideal for treating the domestic waste-water of small cities, townships, and villages, as well as communities in peri-urban areas, which have no sewage pipeline network.

### Intrinsic merits of BPDST

Anaerobic digestion has been broadly recognized as the core of sustainable waste management. BPDSTs are based on a set of treatment principles that ensure treatment reliability, longevity, and tolerance toward inflow fluctuations. BPDSTs work with or without relatively low energy input; thus, the systems cannot be shut down accidentally. Most of the materials/inputs are locally available; thus, BPDST presents an affordable solution that guarantees long-term and reliable operation. Practice has shown that BPDSTs have several advantages compared with centralized sewage plants (Qian et al. 2014). The lack of elaborate sewer systems significantly reduces the investment on operation and maintenance (O&M) costs. BPDSTs can remove organic pollutants, pathogens, and parasite eggs (Sasse 1998; Gutterer et al. 2009) and possibly recover biogas as fuel. BPDSTs could be constructed underground without occupying land. Moreover, BPDSTs have less negative impact on the environment and are suit-able for ecologically sensitive regions (Lu et al. 2009). However, BPDSTs are not always the best solution in every case. Nevertheless, when skilled and responsible O&M cannot be guaranteed, BPDST technologies are undoubtedly the best choice available.

### Government support and policy drive

In 2000, the former Ministry of Construction, former State Environmental Protection Administration (SEPA), and the Ministry of Science and Technology co-released the Technical Policy on Municipal Wastewater Treatment and Pollution Prevention, which explicitly stipulated that dispersed settlements without access to a municipal sewage collection system should adopt onsite wastewater treatment technology and make the treated effluent meet required standards. In 2006, biogas dissemination in China was proposed as part of rural infrastructure for new rural construction for the first time, as stated in the Premier’s Government Working Report (‘Strengthen the rural infrastructure, namely, road, biogas, water, electricity, and communication etc.’). In 2007, SEPA released Opinions on Strengthening Rural Environmental Protection Work, which explicitly specified the reinforcement of pollution control of wastewater in rural areas. Moreover, MOA and the Ministry of Housing and Urban–Rural Development (MOHURD) successively launched a series of guidelines on rural domestic wastewater treatment. Under the instruction of central government, many local regulations and policies were established, which are normally stricter than those issued by the central government. For example, Sichuan Province led in implementing BPDSTs by promulgating a provincial policy. Furthermore, MOA issued a set of standards to guide the design, construction, and operation and maintenance of biogas digesters for domestic sewage treatment (MOA 2009, 2014a, 2014b, 2014c; Table 7). In 2015, the State Council launched the Water Pollution Prevent and Control Action Plan (also named ‘Water 10 Action Plan’), which also emphasized wastewater treatment in rural areas, and the central government appropriated 6 billion CNY for rural environment improvement (MEP 2016).

**Table 7:** Related standards for BPDST in China

Standard no.	Name	Scope	Highlights
NY/T 2597-2014	Collection of standard design drawings of BPDST	It provides the BPDST drawing for optional and combined technology.	The drawings aim at brick–concrete structure of BPDSTs. Three series (Types A, B, and C) should meet different economic and environmental conditions, as well as effluent requirements. The effective volume varies between 20 m3 and 200 m3. Type A adopts combined drainage system; effluent should meet the hygienic requirement for harmless disposal of human waste, which is roundworm mortality _95%; living eggs of Schistosoma and hookworm are undetectable; salmonella is undetectable; and thermotolerant coliforms _10_4. Type B adopts separated drainage system; effluent should meet the grade 3 discharge standard of pollutants for municipal WWTPs. Type C adopts separated drainage system; effluent should meet the grade 2 discharge standard of pollutants for unicipal WWTPs.
NY/T 2601-2014	Construction regulations of BPDST	It sets out the construction procedure and technical requirement for BPDST and suits the newly built, expanded, and rebuilt BPDST but excludes household biogas digesters in rural areas.	Corresponding approval documents should be obtained before construction. Main materials should be qualified and certificated. Pipeline work should follow related standards. Installation of filler should follow related requirements. Water tightness and air-tight test should be done once construction is finished. Local rural energy office is responsible for accepting completed projects, including midterm and final acceptance.
NY/T 2602-2014	O&M specifications of BPDST	It sets out the requirement and methods for O&M of BPDST.	The O&M staff should be trained and certificated. Gas tightness should be checked annually. Overhaul should be conducted every 2–4 years. The effluent should be monitored regularly. Key indexes include pH, COD, BOD, NH4 _–N, TP, TN, and fecal coliform. No need to collect biogas for less than 10 m3 anaerobic digester. Measures should be considered for safety control. Key data, such as drawings, should be documented.

### Reuse demand

China is a water-scarce country. The annual water shortage gap reaches more than 30 billion m^3^ in the agriculture sector, and 60% of cultivated land lacks irrigation water. Consequently, the output of food produce is reduced by 35–40 billion tonnes per year (Li 2012). According to the Water-saving Social Construction 13th Five-Year Plan, the water-saving level in the agriculture sector is still low and water consumption should be controlled (NDRC et al. 2017). If the effluent from BPDSTs can be reused in agriculture, the development of BPDST is beneficial in achieving water-saving targets in the agriculture sector. Waste recovery is desirable not only to reduce pollution but also to improve environmental sustainability (Zhang & Zhang 2008; Zurbrügg & Tilley 2009; Andersson et al. 2016). Anaerobic treatment generates a good use of the resources in wastewater and increases recycling rates. After combining units, wastewater from several flow streams is disposed to reach discharge standards and allow hygienic reuse in agriculture (WHO 2016). Instead of treating waste, a valuable resource is being exploited. Effluent is suitable for surface irrigation. According to the above-mentioned literature review, most sample effluent can meet the national reuse standard for irrigation GB 5084-2005 *Standards for Irrigation Water Quality* (i.e. COD ≤ 200, BOD_5_ ≤ 100, SS ≤ 100, thermotolerant coliforms ≤ 4,000/100 mL, parasitic ovum ≤ 2/L, and pH = 5.5–8.5). Furthermore, most sample effluent can also meet the WHO guidelines for the safe use of wastewater, excreta, and greywater to some extent (WHO 2016). For example, in DPR Korea, reuse is monitored twice per year under WHO irrigation standards. Processed wastewater can be used for fish farming when diluted with fresh river water or after extensive treatment in pond systems. Sludge from BPDSTs can be used to produce organic fertilizer. Theoretically, the removal of each kg of COD may produce 0.35 m^3^ methane (Mang & Li 2010). Organic wastewater can generate a considerable amount of biogas as a substitute for cooking fuel.

## CHALLENGES FOR DEVELOPING BPDST

### Uncertain responsibility

Wastewater treatment in rural areas requires complicated system engineering, which involves several departments, such as construction, environmental protection, agriculture and forestry, and hygiene. When implemented, BPDST faces cross-management from different departments. That is to say, the implementation of BPDST must undergo a series of examinations and approvals, which counteract BPDST dissemination. The function of each department is not clarified; thus, coordination is not smooth. For example, all-level rural energy offices assume the responsibility of ‘utilizing biogas technology and controlling environmental pollution,’ and are thus in charge of R&D and demonstration projects. The functions of construction, supervision, and management belong to the department of construction and the department of environmental protection. In case the concerned departments do not reach an agreement, the distribution of responsibilities is unclear, which results in a lack of coordination. From project plan to project implementation, obstacles are inevitable.

### Uncertain effluent quality

BPDST employs an anaerobic system at its core. However, when it deals with nutrient removal, especially ammonia removal, effluent from BPDSTs does not meet the discharge standard at times if post aerobic treatment is missing. The advantages and disadvantages of anaerobic treatment against aerobic treatment in terms of environment, energy, and ecology are presented in Table 8. In addition, the incoming water flow for decentralized systems fluctuates more than in centralized systems, which may result in an unsteady and uncertain effluent quality.

**Table 8:** Advantages and disadvantages of anaerobic treatment against aerobic treatment

	Anaerobic treatment	Aerobic treatment
Advantages	Energy saving and energy output: It requires no aeration but produces biogas. Ecological benefit: Effluent can be reused as organic fertilizer or for irrigation. Low sludge yield. It can treat some organic matters that cannot be degraded by aerobic technology.	High efficiency of nitrogen and phosphorus removal: It is more likely to achieve the effluent discharge standards than anaerobic systems. Effluent quality is more steady and expectable.
Disadvantages	Limited COD/BOD elimination: Effluent quality is normally worse than aerobic treatment for a certain wastewater. Pathogen removal is limited and typically inferior to aerobic systems. Odor emission. Long retention time and large tank volume. Malfunction of nutrient removal.	High energy consumption: It requires additional energy input for aeration at times. More O&M.

### Low environmental awareness

A survey reveals the public opinion that environmental pollution has become a serious problem for China (Liu et al. 2010). Public awareness toward the problem of wastewater pollution has grown tremendously in recent years. However, the education gap between urban and rural areas is still large. When residents or farmers were interviewed about the BPDST in their area, they had no idea about these structures; to a certain extent, this reflected the attitude of the local people toward the technology. Another issue is public acceptance. Farmers prefer chemical fertilizer instead of organic fertilizer. They expect a payment if they use the ‘waste sludge’ from biogas digesters. This situation shows a great need for dissemination of knowledge about the reuse-oriented wastewater treatment systems.

### Lack of funds and technological innovation

Money is not often the most serious problem for pilot or demonstration projects. However, a general helplessness exists when it comes to individual implementation and more so when it comes to active and well-organized BPDST dissemination. With time, the traditional processes and facilities cannot keep pace with user demand (Gao et al. 2014). R&D investment for new technology should be increased and innovation systems that are market oriented and adopted by research institutions should be established to promote technological progress. R&D should be encouraged, and special funding should be available for it. These funds should be directly allocated to enterprises, such that their capacity for innovation and sense of responsibility are stimulated.

A complete management system for scientific and technological achievements should be built, intellectual property should be safeguarded, and independent innovation should be supported. The appraisal and award system should be conducted to translate the results of scientific research into productive forces effectively.

### Lagging follow-up service and management

BPDST development in rural China focuses mainly on construction and fails to consider management. Thus, a number of biogas projects have been terminated because of a lack of follow-up services and management. At present, when one project is finished, specification for completion acceptance should follow; however, the follow-up service is always neglected. In many areas, once a biogas project is built, no one manages it anymore. Unfortunately, the result of years of neglect is a failing system that can pollute groundwater, as well as nearby lakes and streams. Failing systems also often generate foul odors. Specialized, literate staff is in short supply in rural China, and once out of function, BPDSTs are left unrepaired for a long time. The users or operators do not have to be professional technicians but at least should be trained in the operation and possess the knowledge to maintain BPDSTs

## LESSONS LEARNED AND PERSPECTIVES

Similar systems for rural wastewater management are also developed and introduced in other countries, such as India, Tanzania, Zambia, Thailand, DPR Korea, Indonesia, Afghanistan, Bangladesh, Kenya, Nepal, Rwanda, South Africa, Lesotho, Jordan, Mexico, and Vietnam. These systems are mainly known as DEWATS or DESAR and have had support from Bremen Overseas Research and Development Association, Swiss Development Cooperation, Swiss Federal Institute of Aquatic Science and Technology, Technologies for Economic Development, Ecosan Services Foundation, Gesellschaft für Internationale Zusammenarbeit (German Development Cooperation), Water Services Trust Fund, Consortium for DEWATS Dissemination, Asian Institute of Technology, Biogas Institute of the Chinese Ministry of Agriculture, Environment and Public Health Organization, and Deutsche Welthungerhilfe (German World Hunger Relief). Similar hindrances mentioned above may also be experienced by other countries if such systems are introduced or developed. The selection of BPDST is based on housing den-sity, low or zero energy consumption, reuse options of wastewater nutrients, and avoidance of climate that affects gas emissions by capturing methane. Meanwhile, monetary consideration must never be neglected.

Low efficiency of nitrogen and phosphorus removal restricts BPDST to meet the discharge standard if only anaerobic systems are applied. High treatment efficiency can be reached depending on the post-treatment steps (e.g. sequencing batch reactor (SBR), aerated filter, trickling filter, membrane bio-reactor, constructed wetland, and lagoon). Some of these technologies are already part of the standards. Thus, the effluent from BPDST is reused in irrigation, in which case nutrients require no removal. Approximately 140 billion CNY (ca. 20 billion USD) is reported to be invested in rural wastewater in China during the 13th Five-Year Plan (2016–2020), of which BPDSTs will be a part.

## CONCLUSIONS

BPDST is a promising option for wastewater management in rural China. BPDST development has several opportunities in rural China, including fulfilling the MDGs and SDGs, the water pollution situation and deficiency of wastewater treatment facilities, advantages of BPDST compared with centralized sewage plant, government support and policy drive for rural wastewater treatment, and reuse demand for resources. However, challenges are encountered and should be managed. These challenges include uncertain responsibility of departments, uncertain effluent quality due to low efficiency of nutrient removal, low environmental awareness, lack of funds and technology innovation, and lagging follow-up service and management. Correspondingly, the responsibility of each department should be specified; education and training should be conducted to raise environmental awareness and knowledge of BPDST during dissemination; more funds should be invested in BPDST R&D to improve technology innovation; more reuse elements should be considered during BPDST implementation; and measures should be considered to improve the follow-up service and management of BPDST.
